# PaSTO-GNN: prompt-aware spatio-temporal graph neural networks for automatic essay scoring

**DOI:** 10.3389/frai.2026.1842542

**Published:** 2026-07-08

**Authors:** Areej Alhothali

**Affiliations:** Department of Computer Science, Faculty of Computing and Information Technology, King Abdulaziz University, Jeddah, Saudi Arabia

**Keywords:** automated essay scoring, natural language processing, ordinal regression, prompt-aware learning, spatio-temporal graph neural networks

## Abstract

Automatic Essay Scoring (AES) aims to evaluate the quality of written essays automatically, providing fast, consistent, and objective assessments of students' writing ability. Existing deep learning approaches—including recurrent, convolutional, and transformer-based models—primarily focus on textual semantics, yet they often overlook the spatio-temporal nature of essay composition, where meaning evolves across sentences and paragraphs through discourse progression. To address this gap, this study presents a prompt-aware Spatio-Temporal Graph Neural Network (PaSTO-GNN) for AES. In this framework, each essay is first segmented into sentences, and each sentence is represented as a node in a spatio-temporal graph. The feature representation of each node is constructed by combining contextual sentence embeddings extracted from a RoBERTa encoder adapted via Low-Rank Adaptation (LoRA), semantic embeddings obtained from Sentence-BERT (SBERT), and a learned prompt embedding that conditions scoring on the essay prompt. Spatial edges capture semantic relationships between sentences, while temporal edges encode the sequential progression of ideas throughout the essay. The resulting node representations are processed through a spatio-temporal message passing network, followed by a BiGRU layer and temporal attention pooling to obtain a global essay representation. To model the ordered nature of essay scores, prompt-specific ordinal prediction heads based on CORAL are employed, together with a per-prompt calibration step that better aligns predicted scores with human scoring distributions. Experimental results on the AES 2.0 benchmark dataset show that PaSTO-GNN achieves a Quadratic Weighted Kappa (QWK) of 0.8329 on the validation set after prompt calibration and a Pearson correlation of 0.8168, highlighting the effectiveness of combining spatio-temporal discourse modeling with prompt-aware representations for automated essay evaluation.

## 1 Introduction

The rapid growth of online education platforms has significantly increased the demand for scalable and automated assessment tools. In modern learning environments, assessment plays a crucial role in evaluating students' skills, abilities, and learning needs. Most online examination systems rely on automatically graded question formats such as multiple-choice or short-answer questions. However, automatically evaluating essays remains a challenging problem due to the complexity and subjectivity involved in assessing written language.

Essay scoring is widely used to evaluate students' writing ability and language proficiency. Language ability is generally described through three main components: linguistic competence, discourse competence, and sociolinguistic competence (Hymes, 1972; Canale and Swain, 1980; Bachman, 1990). Linguistic competence refers to knowledge of grammar, vocabulary, and the structural aspects of language. Discourse competence focuses on the ability to organize ideas coherently and maintain cohesion across sentences and paragraphs. Sociolinguistic competence involves producing language that is appropriate for a specific audience and communicative context. Together, these competencies form the foundation for evaluating writing ability in many educational assessment frameworks.

Similar criteria are commonly used to evaluate the writing ability of English as a Foreign Language (EFL) learners and other English language learners. In writing assessments, learners are typically given a prompt describing a particular issue or topic that they must address in an essay. Essays are then evaluated based on multiple criteria reflecting different aspects of writing quality.

In practice, written essays are commonly assessed using three types of scoring scales: primary trait scales, holistic scales, and analytic scales (Weigle, 2002). Primary trait scoring evaluates how effectively an essay fulfills a specific writing task or communicative purpose defined by the prompt. Holistic scoring assigns a single overall score based on the general quality of the essay without evaluating individual components separately. In contrast, analytic scoring scales provide detailed evaluation across multiple dimensions of writing, such as content relevance, organization, coherence and cohesion, vocabulary adequacy, grammar, and mechanics. Table 1 illustrates an example of an analytic scoring scale used for evaluating writing performance.

**Table 1:** Example of analytic writing assessment scales (Cumming, 2008).

Category	Low performance	High performance
Relevance and adequacy of content	The answer bears almost no relation to the task set	Relevant and adequate response to the task set
Compositional organization	No apparent organization of content	Overall structure and internal organization are clear
Cohesion	Cohesion almost totally absent	Effective use of cohesive devices enabling clear communication
Adequacy of vocabulary for purpose	Vocabulary inadequate even for basic communication	Vocabulary is appropriate and adequate for the task
Grammar	Almost all grammatical patterns inaccurate	Almost no grammatical inaccuracies
Mechanic (punctuation)	Ignorance of punctuation conventions	Almost no inaccuracies in punctuation
Mechanic (spelling)	Almost all spelling inaccurate	Almost no inaccuracies in spelling

Although analytic scoring provides a detailed evaluation of writing quality, manually assessing essays is time-consuming, costly, and difficult to scale in large educational settings. This challenge has motivated increasing research interest in Automatic Essay Scoring (AES), which aims to automatically evaluate the quality of written essays using computational techniques.

To automatically evaluate written essays, researchers have investigated natural language processing and machine learning techniques to analyze essays and produce scores that reflect the quality of the written text. This line of research has introduced the field of Automatic Essay Scoring (AES) (Beseiso et al., 2021). AES systems aim to automatically assign grades to essays written in educational settings, enabling fast, consistent, and scalable assessment. Since the early development of AES systems in 1973 (Ajay et al., 1973), researchers have explored various computational approaches for evaluating written essays. Recent advances in computational power and neural network technologies have further accelerated the development of intelligent natural language processing systems for educational assessment.

The AES task is commonly formulated as a supervised learning problem, either as a classification task that assigns essays to discrete score categories or as a regression task that predicts continuous scores. Early AES systems relied heavily on hand-crafted linguistic and statistical features, making their performance highly dependent on the quality of feature engineering. Typical features include sentence length, vocabulary richness, grammar usage, and text similarity to reference responses. These features were commonly used with machine learning algorithms such as linear regression (Sultan et al., 2016), support vector machines (Persing and Ng, 2013; Ke et al., 2019), and gradient boosting methods such as XGBoost (Salim et al., 2019).

With the emergence of deep learning, neural network models capable of automatic feature extraction have become widely adopted in AES research. Instead of relying on manually engineered features, these approaches learn semantic representations directly from text using word or character embeddings. Several studies explored convolutional neural networks (CNN) (Dong and Zhang, 2016), recurrent neural networks such as Long Short-Term Memory (LSTM) (Riordan et al., 2017; Dong et al., 2017), and Bidirectional LSTM models (Taghipour and Ng, 2016; Wang Y. et al., 2018; Zhu and Sun, 2020). More recently, attention mechanisms and transformer-based models such as BERT and RoBERTa have demonstrated strong performance in AES tasks (Rodriguez et al., 2019). These models have achieved competitive results compared to traditional machine learning approaches by effectively capturing contextual semantic information. In addition, several hybrid approaches have been proposed to combine statistical features with contextual representations to further improve scoring performance (Zhu and Sun, 2020).

In recent years, Graph Neural Networks (GNNs) have attracted increasing attention in machine learning and natural language processing tasks (Yao et al., 2019). GNNs are particularly effective for modeling structured data and relational dependencies. They have been successfully applied in various predictive analytics tasks and have shown competitive performance compared with traditional machine learning and deep learning approaches (Kipf and Welling, 2017; Wu et al., 2020; Zhou et al., 2020). In NLP applications, GNNs allow text to be represented as graphs in which words, sentences, or documents are modeled as nodes and their relationships as edges, enabling the capture of complex structural dependencies (Huang et al., 2019).

Recent advances in GNNs have shown promise in modeling relational structures in natural language. By representing words, sentences, or documents as nodes and their relationships as edges, GNNs can encode complex dependencies beyond sequential order. Some studies have applied GNN-based methods to AES by constructing static graphs using word co-occurrence or syntactic dependencies. However, these approaches remain spatially limited, as they ignore how discourse evolves across sentences and fail to explicitly model the temporal dynamics of essay development.

To address this limitation, we propose a prompt-aware Spatio-Temporal Graph Neural Network (PaSTO-GNN) for Automatic Essay Scoring. In this framework, essays are represented as spatio-temporal graphs in which nodes correspond to sentence-level semantic units and edges capture both spatial dependencies (semantic relationships between sentences) and temporal progression (the sequential development of ideas). Each node is initialized using complementary sentence representations that combine contextual embeddings extracted from a RoBERTa encoder adapted via Low-Rank Adaptation (LoRA) with semantic embeddings obtained from Sentence-BERT (SBERT), together with a learned prompt embedding that conditions scoring on the essay prompt. Through spatio-temporal message passing and sequential refinement, the model learns discourse-aware essay representations that capture both structural coherence and semantic relationships across sentences.

Based on this framework, our study makes the following key contributions:

- **Prompt-aware spatio-temporal graph architecture for AES:** we propose PaSTO-GNN, a prompt-aware spatio-temporal graph neural network designed for automatic essay scoring. The model explicitly captures spatial relationships among sentences while simultaneously modeling the temporal progression of discourse throughout the essay.
- **Multi-embedding sentence representation:** the framework incorporates complementary sentence representations by combining contextual embeddings obtained from a RoBERTa encoder adapted using Low-Rank Adaptation (LoRA) with semantic sentence embeddings generated by Sentence-BERT (SBERT). In addition, learned prompt embeddings are introduced to condition the scoring process on the specific essay prompt.
- **Discourse-aware spatio-temporal modeling:** the proposed method constructs spatio-temporal graphs in which spatial edges represent semantic relations between sentences, while temporal edges capture the sequential development of ideas. This design enables the model to learn discourse-level coherence and structural organization within essays.
- **Ordinal score prediction with calibration:** considering the discrete and ordered nature of essay scores, the framework adopts prompt-specific ordinal regression using CORAL. A *post-hoc* calibration step is further applied for each prompt to better align model predictions with the distribution of human-assigned scores.
- **Empirical validation:** experimental evaluation on the AES 2.0 benchmark datasets shows that the proposed approach achieves strong performance in terms of Quadratic Weighted Kappa (QWK) and Pearson correlation when compared with several competitive neural baselines.

Overall, this research highlights the importance of spatio-temporal discourse modeling and prompt-aware learning in essay assessment and introduces a unified framework that improves the robustness and generalization of AES systems.

## 2 Related work

Automatic Essay Scoring (AES) has evolved significantly over the past two decades, progressing from feature-engineering approaches to deep neural architectures and, more recently, transformer- and large language model (LLM)-based systems. Early AES research primarily relied on handcrafted linguistic and statistical features such as grammar usage, vocabulary richness, syntactic complexity, and discourse indicators. Systems such as e-rater and IntelliMetric demonstrated that regression and tree-based models could approximate human scoring with reasonable agreement (Attali and Burstein, 2006; Rudner and Liang, 2002). Subsequent studies incorporated machine learning algorithms, including Support Vector Machines (SVM), Random Forests, and gradient boosting, achieving improved performance on benchmarks such as the ASAP Hewlett Foundation dataset (Phandi et al., 2015; Dong et al., 2017). For example, (Salim et al. 2019) proposed an XGBoost classifier using statistical features such as word count, part-of-speech tags, parse tree depth, and sentence similarity percentages. Similarly, (Ke et al. 2019) proposed a support vector machine–based method to score individual essay quality dimensions and then aggregate them to produce the final score. Despite their interpretability, these approaches suffered from limited generalization and heavy reliance on manually designed features.

With the rapid advancement of deep learning in natural language processing, neural network–based AES methods emerged as a promising alternative to feature-engineering approaches. These models automatically learn semantic representations from text, eliminating the need for manual feature design. Convolutional Neural Networks (CNN) and Recurrent Neural Networks (RNN) were widely adopted for modeling essay semantics and structure (Taghipour and Ng, 2016). For instance, (Cai 2019) introduced a recurrent neural network model for AES and demonstrated that deep learning approaches outperform traditional feature-engineering methods. (Riordan et al. 2017) proposed a hybrid CNN-LSTM model trained on word embeddings to capture both local and sequential textual patterns. Similarly, (Riordan et al. 2019) explored character-level embeddings to address spelling errors in student essays. Long Short-Term Memory (LSTM) networks were particularly effective in modeling long-range dependencies and discourse flow (Alikaniotis et al., 2016). Hybrid architectures combining CNN and LSTM layers further improved performance by capturing both local textual features and global essay structure.

Several studies also investigated hierarchical neural architectures to model the multi-level structure of essays. For example, (Dong and Zhang 2016) proposed a hierarchical CNN model that separately represents sentence-level and essay-level structures. Later, (Dong et al. 2017) introduced a hierarchical recurrent network with attention mechanisms to learn essay representations automatically. Their work compared word embeddings, character embeddings, and hybrid representations, demonstrating improved scoring performance. Other neural models explored various architectures, including Bidirectional LSTM networks (Wang Y. et al., 2018; Wang Z. et al., 2018), Siamese BiLSTM frameworks for learning text semantics (Liang et al., 2018), and reinforcement learning approaches that directly optimize the Quadratic Weighted Kappa (QWK) evaluation metric (Wang Y. et al., 2018). In addition, neural models with cross-sentence dependencies were developed for datasets such as the TOEFL essay corpus to better capture discourse relationships across sentences (Nadeem et al., 2019). More recent work has extended this line of research through contrastive and cross-prompt learning paradigms. For example, (Xie et al. 2022) proposed pairwise contrastive regression for AES, while (Chen and Li 2023) introduced prompt-mapping contrastive learning for cross-prompt essay scoring. Likewise, (Chen and Li 2024) developed a prompt-generalized and level-aware framework for cross-prompt AES, highlighting the continued importance of prompt sensitivity and score transferability. Overall, these studies demonstrated the effectiveness of deep neural networks in capturing semantic and structural information in essays.

The introduction of pretrained language models further advanced AES research by enabling contextualized text representations. Word embedding methods such as GloVe, ELMo, and BERT were widely investigated for essay scoring tasks. For example, (Wangkriangkri et al. 2020) compared AES models using GloVe, ELMo, and BERT embeddings and found that BERT-based models achieved the most robust performance. Similarly, (Cozma et al. 2018) proposed a hybrid AES approach that combines string kernels with word embeddings, achieving strong scoring performance. Transformer-based models such as BERT, RoBERTa, and XLNet further improved contextual language understanding and became dominant approaches in AES systems (Rodriguez et al., 2019). More recent studies continued this trend by using BERT-based multi-scale essay representations and transformer fine-tuning strategies to improve scoring robustness across prompts and score ranges ?. However, the computational complexity of large transformer models has raised concerns regarding efficiency and scalability (Ormerod et al., 2021). To address this issue, (Ormerod et al. 2021) investigated lightweight transformer models and demonstrated that competitive AES performance can be achieved with smaller parameter sizes.

More recently, AES research has expanded toward large language models and prompt-based scoring. (Mansour et al. 2024) examined whether LLMs such as ChatGPT and Llama can automatically score essay proficiency, while (Lee et al. 2024) proposed a zero-shot prompting framework for essay scoring based on multi-trait specialization. In a related study, (Pack et al. 2024) analyzed the validity and reliability of several prominent LLMs, including GPT-3.5, GPT-4, Claude 2, and PaLM 2, for scoring English language learner essays. Similarly, (Li and Liu 2024) explored LLM-based AES for non-native Japanese writing. Although these studies demonstrate the strong potential of LLMs for AES, they also raise concerns regarding score consistency, interpretability, and dependence on prompting strategies.

Several studies have also explored hybrid AES frameworks that combine statistical and semantic features. For example, (Zhu and Sun 2020) proposed a multimodal neural architecture that integrates statistical features with semantic contextual representations. Their approach uses an LSTM model with GloVe embeddings for textual representation while another neural network processes statistical features, and the outputs are combined to predict the final essay score.

In addition to sequential and transformer-based models, recent studies have explored graph neural networks (GNNs) to capture structural relationships in textual data. GNNs have demonstrated strong performance in various machine learning and NLP tasks due to their ability to model relational structures (Kipf and Welling, 2017; Wu et al., 2020; Zhou et al., 2020). In text analysis, documents can be represented as graphs where words, sentences, or documents are modeled as nodes connected through semantic or syntactic relationships (Huang et al., 2019). For AES tasks, graph-based approaches enable explicit modeling of coherence and inter-sentence dependencies that are difficult to capture using purely sequential architectures. Recent structure-aware AES studies have moved in this direction. For example, (Yamaura et al. 2023) incorporated logical structure into neural AES, and (Kato 2025) further explored graph attention over logical-structure graphs for essay scoring. Recent work has also explored hybrid architectures that integrate Transformer representations with graph neural networks (Aljuaid et al. 2025). These studies confirm the value of explicit structural modeling; however, many existing GNN-based AES methods still rely on static graph structures that capture spatial relationships while overlooking the temporal progression of ideas within essays.

Therefore, despite substantial progress in neural, transformer, and graph-based AES, effectively modeling both inter-sentence relationships and the sequential evolution of discourse remains an open challenge. This limitation motivates the development of spatio-temporal graph architectures that can jointly capture semantic connectivity and discourse progression within essays.

## 3 Methodology

### 3.1 Overview

We propose PaSTO-GNN (Prompt-aware Spatio-Temporal Ordinal Graph Neural Network), a graph-based framework for Automatic Essay Scoring (AES) that models an essay as an ordered set of sentence-level nodes. The model integrates contextual sentence representations from a RoBERTa encoder adapted with Low-Rank Adaptation (LoRA), semantic sentence embeddings from Sentence-BERT (SBERT), and a learned prompt embedding to explicitly condition scoring on the essay prompt.

The fused sentence representations are organized into a spatio-temporal graph where temporal edges capture discourse order and spatial edges capture semantic similarity between sentences. A spatio-temporal message passing network propagates information across this graph, followed by sequential refinement using a Bidirectional GRU and temporal attention pooling to produce an essay-level representation.

**Figure 1:**
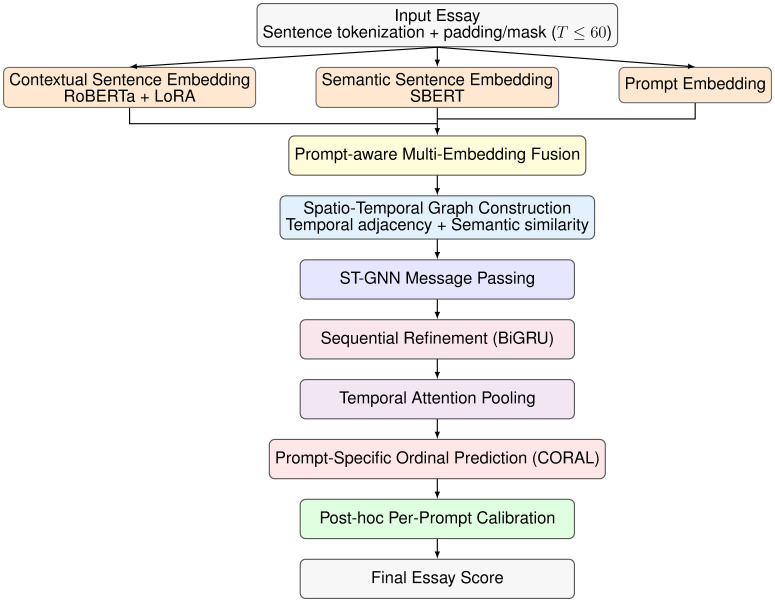
Architecture of the proposed PaSTO-GNN framework for automatic essay scoring.

Finally, essay scores are predicted using prompt-specific ordinal prediction heads based on the CORAL framework, and a *post-hoc* calibration step is applied to better align predictions with human scoring distributions. The overall architecture of the proposed PaSTO-GNN framework is illustrated in Figure 1.

### 3.2 Prompt-aware sentence representation with multi-embedding fusion

Given an essay *E* = {*s*_1_, …, *s*_*T*_}, each sentence *s*_*t*_ is represented using three complementary components:

- **Trainable contextual embedding** $x_{t}^{\text{ctx}}\in {\mathbb{R}}^{d_{c}}$ obtained from a RoBERTa encoder. We adapt RoBERTa using Low-Rank Adaptation (LoRA) within the parameter-efficient fine-tuning (PEFT) framework by injecting low-rank updates into the attention query and value projections.
- **Fixed semantic embedding** $x_{t}^{\text{sbert}}\in {\mathbb{R}}^{d_{s}}$ obtained from SBERT (all-MiniLM-L6-v2), computed without gradient updates.
- **Prompt embedding** $e_{p}\in {\mathbb{R}}^{d_{p}}$, a learned embedding associated with prompt *p*, which is broadcast across the *T* sentence positions to condition scoring on the essay prompt.

The final node representation is constructed by concatenating these components, as shown in Equation 1:


$$\begin{array}{l} x_{t}=x_{t}^{\text{ctx}}∥x_{t}^{\text{sbert}}∥e_{p}. \end{array}$$ (1)

A fusion multilayer perceptron (MLP) then projects *x*_*t*_ into a shared latent space, as shown in Equation 2:


$$\begin{array}{l} h_{t}^{\left(0\right)}=\text{ReLU}\left(W_{f}x_{t}+b_{f}\right), \end{array}$$ (2)

followed by dropout regularization. The resulting masked node representation matrix is denoted by *H*^(0)^∈ℝ^*T*×*d*^.

### 3.3 Spatio-temporal graph construction

To model both the sequential structure and semantic relationships within essays, we construct two adjacency matrices for each essay: a temporal adjacency matrix *A*^(*t*)^ and a spatial adjacency matrix *A*^(*s*)^. The temporal graph captures the natural progression of sentences in the essay, while the spatial graph captures semantic similarity between sentences that may be distant in the text.

**Temporal adjacency**. Temporal edges connect consecutive sentences in order to preserve the discourse sequence of the essay. The temporal adjacency matrix is defined in Equation 3. Specifically,


$$A_{ij}^{\left(t\right)}=\{\begin{array}{ll} 1, & j=i+1\text{ or }i=j+1,\\ 0, & \text{otherwise}. \end{array}$$ (3)

This construction links each sentence to its immediate neighbors, enabling the model to capture local discourse flow. Padded nodes introduced during batching are removed through masking, and the adjacency matrix is row-normalized according to node degree.

**Spatial adjacency**. In addition to sequential relations, essays may contain semantically related sentences that are not adjacent in the text. To capture such relationships, we construct spatial edges using cosine similarity computed from SBERT sentence embeddings, as shown in Equation 4:


$$\begin{array}{l} \text{sim}\left(i,j\right)=\frac{\langle x_{i}^{\text{sbert}},x_{j}^{\text{sbert}}\rangle }{\left||x_{i}^{\text{sbert}}||||x_{j}^{\text{sbert}}|\right|}. \end{array}$$ (4)

An edge is retained if the similarity exceeds a threshold τ_*s*_ and the sentence belongs to the **top-k** most similar neighbors of node *i*, as shown in Equation 5:


$$A_{ij}^{\left(s\right)}=\{\begin{array}{ll} \text{sim}(i,j), & \text{sim}(i,j)\geq \tau_{s}\;\wedge \;j\in \text{TopK}(i),\\ 0, & \text{otherwise}. \end{array}$$ (5)

Self-loops are added for valid nodes to preserve their original representations, and the resulting matrix is row-normalized.

### 3.4 Spatio-temporal message passing

Given the constructed spatial and temporal graphs, we apply *L* spatio-temporal message passing layers to jointly aggregate information from both semantic and sequential neighborhoods, as shown in Equations 6–8. At layer ℓ, spatial and temporal messages are first computed as:


$$\begin{array}{l} M_{s}^{\left(\ell \right)}=A^{\left(s\right)}H^{\left(\ell \right)},\;M_{t}^{\left(\ell \right)}=A^{\left(t\right)}H^{\left(\ell \right)}, \end{array}$$ (6)

where *H*^(ℓ)^ denotes the node representations at layer ℓ. The aggregated messages are then combined through learnable transformations:


$$\begin{array}{l} {\tilde{H}}^{\left(\ell +1\right)}=\text{ReLU}\left(W_{s}M_{s}^{\left(\ell \right)}+W_{t}M_{t}^{\left(\ell \right)}+b^{\left(\ell \right)}\right). \end{array}$$ (7)

To stabilize training and preserve previously learned representations, dropout and a residual connection with layer normalization are applied:


$$\begin{array}{l} H^{\left(\ell +1\right)}=\text{LayerNorm}\left(H^{\left(\ell \right)}+{\tilde{H}}^{\left(\ell +1\right)}\right). \end{array}$$ (8)

All computations are performed with masking to ensure that padded sentences introduced during batching do not influence the graph propagation process.

### 3.5 Sequential refinement with BiGRU

To further capture fine-grained sequential dependencies among sentence nodes, we apply a bidirectional GRU (BiGRU) over the graph-updated node representations. Given the output of the final spatio-temporal layer *H*^(*L*)^, the BiGRU produces contextualized representations:


$$\begin{array}{l} Z=\text{BiGRU}\left(H^{\left(L\right)}\right). \end{array}$$ (9)

Since the BiGRU produces concatenated forward and backward hidden states, we project the output back to the graph latent dimension:


$$\begin{array}{l} \bar{H}=W_{g}Z+b_{g}. \end{array}$$ (10)

This refinement step allows the model to capture sequential dependencies that may not be fully captured by graph propagation alone.

### 3.6 Temporal attention pooling

To obtain an essay-level representation, we apply temporal attention over the sequence of sentence representations. The attention weights are computed using Equation 11:


$$\begin{array}{l} \alpha_{t}=\frac{\exp u^{⊤}\tanh\left(W_{a}{\bar{h}}_{t}\right)}{\overset{T}{\underset{k=1}{\sum }}\exp u^{⊤}\tanh\left(W_{a}{\bar{h}}_{k}\right)}, \end{array}$$ (11)

and the final essay representation is obtained by a weighted aggregation of sentence nodes, as shown in Equation 12:


$$\begin{array}{l} h_{E}=\overset{T}{\underset{t=1}{\sum }}\alpha_{t}{\bar{h}}_{t}, \end{array}$$ (12)

where attention logits corresponding to padded positions are masked to prevent them from contributing to the final representation.

### 3.7 Prompt-specific ordinal prediction with CORAL

Essay scores are inherently ordinal, and different prompts exhibit different score ranges. For each prompt *p*, we define the number of score levels as *K*_*p*_ = *h*_*p*_−ℓ_*p*_+1. To model the ordered structure of labels, we employ the CORAL (COntinuous RAnk Logits) framework, which predicts *K*_*p*_−1 ordered binary thresholds.

Because prompts have varying score ranges, we define a global maximum $K_{\max}=\underset{p}{\max}K_{p}$ and allocate prediction heads that output *K*_max_−1 logits. During training, thresholds exceeding the valid range for a given prompt are masked.

For an essay belonging to prompt *p*, the model outputs logits:


$$\begin{array}{l} z\in {\mathbb{R}}^{K_{\max}-1}. \end{array}$$ (13)

Ordinal targets $T\in {\left\{0,1\right\}}^{K_{\max}-1}$ and a validity mask $M\in {\left\{0,1\right\}}^{K_{\max}-1}$ are constructed such that only the first *K*_*p*_−1 thresholds contribute to the loss. The CORAL loss is defined in Equation 14:


$$\begin{array}{l} L_{\text{CORAL}}=\frac{\underset{k}{\sum }M_{k}\cdot \text{BCEWithLogits}\left(z_{k},T_{k}\right)}{\underset{k}{\sum }M_{k}}. \end{array}$$ (14)

In addition, we compute the expected continuous score from CORAL probabilities and include a small auxiliary regression term, as shown in Equation 15:


$$\begin{array}{l} {ŷ}_{\text{cont}}=\ell_{p}+\underset{k}{\sum }\sigma \left(z_{k}\right)M_{k},\;L_{\text{MSE}}=||{ŷ}_{\text{cont}}-y|{|}_{2}^{2}. \end{array}$$ (15)

The final training objective combines ordinal and regression terms, as defined in Equation 16:


$$\begin{array}{l} L=L_{\text{CORAL}}+\lambda L_{\text{MSE}}, \end{array}$$ (16)

where λ = 0.08 ensures that ordinal learning remains the dominant objective.

### 3.8 *Post-hoc* per-prompt calibration

To better align predictions with prompt-specific scoring distributions, we apply a post-hoc calibration step on the validation set. For each prompt *p*, we search for optimal affine transformation parameters (*a*_*p*_, *b*_*p*_) together with a rounding offset, as defined in Equation 17, that maximize agreement with human scores.


$$\begin{array}{l} {ŷ}^{\prime }=\text{clip}\text{round}\left(a_{p}{ŷ}_{\text{cont}}+b_{p}+o_{p}\right),\;\ell_{p},h_{p}, \end{array}$$ (17)

where the parameters (*a*_*p*_, *b*_*p*_, *o*_*p*_) are selected to maximize Quadratic Weighted Kappa (QWK) on validation essays of prompt *p*. The calibrated mapping is then stored and applied during inference.

## 4 Experiments and results

### 4.1 Datasets

We evaluate the proposed PaSTO-GNN model on the benchmark Automated Student Assessment Prize (AES 2.0) dataset. The dataset contains essays written in response to multiple prompts with heterogeneous scoring ranges. In this work, we use the train_sourcetexts.csv split.

Each essay is segmented into sentences using the NLTK sentence tokenizer. The resulting sentence sequence is truncated or padded to a maximum length of 60 sentences to ensure consistent input size across essays. A binary mask is applied to ignore padded sentences during model training and inference.

To ensure fair evaluation, the dataset is divided into 80% training and 20% validation sets using prompt-stratified sampling. This strategy preserves the distribution of prompts across splits and prevents prompt leakage between training and validation sets. Prompt-specific score ranges (ℓ_*p*_, *h*_*p*_), where ℓ_*p*_ and *h*_*p*_ denote the minimum and maximum scores for prompt *p*, are computed using only the training data and are used to define the ordinal label space for each prompt.

### 4.2 Evaluation metrics

Model performance is evaluated using Quadratic Weighted Kappa (QWK), Pearson Correlation Coefficient (PCC), and Root Mean Squared Error (RMSE). Quadratic Weighted Kappa (QWK), Equation 18, is used as the primary evaluation metric since it measures the level of agreement between predicted essay scores and human ratings while accounting for the ordinal nature of essay scoring.


$$\begin{array}{l} \kappa =1-\frac{\overset{K-1}{\underset{i=0}{\sum }}\overset{K-1}{\underset{j=0}{\sum }}W_{i,j}O_{i,j}}{\overset{K-1}{\underset{i=0}{\sum }}\overset{K-1}{\underset{j=0}{\sum }}W_{i,j}E_{i,j}}, \end{array}$$ (18)

where *O*_*i, j*_ denotes the observed agreement matrix representing the number of essays with true score *i* predicted as score *j*, and *E*_*i, j*_ denotes the expected agreement matrix assuming random agreement between predictions and ground truth. The weight matrix *W* penalizes disagreements according to the distance between score levels and is defined in Equation 19:


$$\begin{array}{l} W_{i,j}=\frac{{\left(i-j\right)}^{2}}{{\left(K-1\right)}^{2}}, \end{array}$$ (19)

where *K* denotes the number of possible score categories.

In addition to the overall QWK, we report per-prompt QWK to analyze the model's performance across different essay prompts in the AES 2.0 dataset. During training, QWK is used for checkpoint selection, learning-rate scheduling, early stopping, and *post-hoc* calibration.

### 4.3 Baselines and state-of-the-art comparisons

We compare the proposed PaSTO-GNN framework with two baseline models: BERT-base and RoBERTa-base. Both baselines are essay-level encoder models in which the entire essay is encoded using the corresponding pretrained transformer. The resulting representation is aggregated using masked mean pooling, combined with a prompt embedding, and passed to prompt-specific CORAL prediction heads for ordinal score prediction. All baseline models are trained using the same training-validation split, prompt-specific score ranges, and evaluation metrics as the proposed model to ensure a fair comparison.

### 4.4 Implementation details

The PaSTO-GNN model is implemented in PyTorch. Each sentence is encoded using two complementary encoders: (i) a roberta-base encoder adapted with Low-Rank Adaptation (LoRA) to obtain trainable contextual sentence embeddings, and (ii) a fixed Sentence-BERT encoder (all-MiniLM-L6-v2) to obtain semantic sentence embeddings. A learned prompt embedding is incorporated to condition the model on the essay prompt. Spatial edges are constructed using cosine similarity between SBERT sentence embeddings with a similarity threshold τ_*s*_ = 0.52 and top-k pruning (*k* = 8). The spatial graph construction parameters were selected empirically through preliminary validation experiments. Lower thresholds produced overly dense graphs with noisy semantic relations, whereas larger thresholds resulted in sparse and partially disconnected graph structures. Similarly, the top-k constraint was introduced to preserve the most semantically relevant sentence relationships while controlling graph complexity and computational cost.

Essay score prediction is formulated as prompt-specific ordinal classification using CORAL with masked thresholds per prompt. The training objective combines CORAL loss with a small auxiliary regression loss on the expected score (λ = 0.08). Optimization is performed using AdamW with separate learning rates for the main model (2 × 10^−4^) and LoRA parameters (8 × 10^−5^). Training uses mixed precision (AMP), gradient clipping with maximum norm 1.0, ReduceLROnPlateau learning-rate scheduling based on validation QWK, and early stopping with a patience of five epochs. We use a batch size of four with gradient accumulation over 2 steps, resulting in an effective batch size of 8. In addition, the LoRA parameters are frozen during the first training epoch and unfrozen thereafter. After training, post-hoc per-prompt calibration is applied to further improve agreement with human scores.

For reproducibility, the proposed framework uses two spatio-temporal graph layers followed by a single-layer BiGRU sequential refinement module. Hyperparameters, including the graph similarity threshold, top-*k* neighborhood size, learning rates, and dropout configuration, were selected empirically through preliminary validation experiments. Training was conducted using PyTorch with mixed precision (AMP) on an NVIDIA GPU environment. Additional implementation details, including optimization settings, gradient accumulation strategy, and training configuration, are provided in this section. The code and implementation details will be made publicly available upon acceptance to support reproducibility and future research.

### 4.5 Experimental results

Table 2 presents the overall validation performance of the proposed model and the baseline approaches on the AES 2.0 dataset. The proposed PaSTO-GNN framework achieves the best performance across all evaluation metrics.

**Table 2:** Overall validation performance on AES 2.0 using a prompt-stratified 80/20 split.

Model	QWK	PCC	RMSE	Cal-QWK
BERT-base	0.7794	0.7821	0.6602	0.7953
RoBERTa-base	0.7828	0.7910	0.6692	0.8041
PaSTO-GNN (raw, best epoch = 9)	**0.8144**	**0.8168**	**0.6190**	**0.8329**

Bold values indicate the best performance among the compared models for each evaluation metric.

Compared with the strongest baseline, RoBERTa-base, the proposed model improves the raw QWK from 0.7828 to 0.8144 and the calibrated QWK from 0.8041 to 0.8329. In addition, it achieves the highest PCC (0.8168) and the lowest RMSE (0.6190), indicating stronger agreement with human raters and lower prediction error.

Table 3 further reports the per-prompt validation QWK scores. The proposed framework achieves the strongest or highly competitive performance across all prompts, demonstrating stable generalization across heterogeneous essay topics and scoring distributions. The largest improvements are observed for prompts such as *Car-free cities* and *Driverless cars*, where the proposed framework substantially outperforms both BERT-base and RoBERTa-base. This observation suggests that incorporating sentence-level graph modeling and sequential contextual refinement can provide complementary discourse-aware information beyond standard essay-level Transformer encoding.

**Table 3:** Per-prompt validation QWK on the AES 2.0 dataset.

Prompt	Samples	BERT-base	RoBERTa-base	Proposed model	Improvement (%)
A Cowboy Who Rode the Waves	435	0.6159	0.6522	**0.7157**	9.74
Car-free cities	392	0.7205	0.7204	**0.8041**	11.60
Does the electoral college work?	409	0.7740	0.7949	**0.8246**	3.74
Driverless cars	1234	0.7396	0.7661	**0.8020**	4.69
Exploring Venus	896	0.8142	0.7940	**0.8173**	0.38
Facial action coding system	977	0.7965	0.8009	**0.8345**	4.20
The Face on Mars	603	0.7391	0.7290	**0.7575**	2.49

Bold values indicate the best performance among the compared models for each evaluation metric.

In contrast, prompts such as *Exploring Venus* show comparatively smaller improvements over the baselines, indicating that certain prompts may rely more heavily on local semantic understanding than on complex discourse structure. Nevertheless, the proposed framework consistently maintains competitive performance across all prompts without significant degradation, highlighting the robustness of the prompt-aware modeling strategy.

Overall, the comparative analysis suggests that combining contextual Transformer representations with discourse-aware sequential and graph-based modeling provides an effective strategy for holistic automated essay scoring. The results further indicate that prompt-aware adaptation and sequential contextual refinement contribute substantially to AES performance in modern benchmark datasets such as AES 2.0.

To evaluate the effectiveness of the proposed PaSTO-GNN framework relative to recent state-of-the-art AES systems, we included a comparison with three recent approaches evaluated on the AES 2.0 dataset, as summarized in Tables 4, 5. AES 2.0 is a more recent and challenging benchmark than ASAP, designed to address several limitations of earlier AES datasets, including limited prompt diversity, restricted essay variability, and reduced realism in modern educational writing assessment scenarios.

**Table 4:** Comparison of Recent AES Studies implemented on AES 2.0 and PaSTO-GNN.

Study	Method	Dataset	Reported QWK
GDBERT-score (Mi and Yu, 2026)	DeBERTa + GCN semantic graphs	AES 2.0	0.7777
ET-GNN (Aljuaid et al., 2025)	Ensemble Transformer-GCN framework	AES 2.0	0.829 (Reg.), 0.841 (Cls.)
SetFit AES (Krug et al., 2025)	SetFit with Longformer sentence transformer	AES 2.0	0.7823
**PaSTO-GNN (proposed)**	Prompt-aware ST-GNN with RoBERTa and BiGRU	AES 2.0	0.8329

**Table 5:** Comparison of modeling and training configurations in recent AES approaches evaluated on AES 2.0.

Study	Word embedding	Graph-based	Sentence-level	Prompt-aware	Same-prompt	Prediction head
GDBERT-score (Mi and Yu, 2026)	DeBERTa	✓	✓	×	✓	Regression
ET-GNN (Aljuaid et al., 2025)	DistilBERT/ RoBERTa/ DeBERTaV3	✓	×	×	✓	Regression/ classification
SetFit AES (Krug et al., 2025)	Longformer	×	✓	×	✓	Classification
**PaSTO-GNN (proposed)**	SBERT + RoBERTa	✓	✓	✓	✓	Ordinal CORAL

The baseline approaches considered in this comparison include: (1) GDBERT-Score (Mi and Yu, 2026), a hybrid DeBERTa-GCN framework that constructs sentence-level semantic graphs using cosine similarity between sentence embeddings; (2) ET-GNN (Aljuaid et al., 2025), an ensemble Transformer-GCN framework combining DistilBERT, RoBERTa, and DeBERTaV3 representations with graph neural networks for holistic essay scoring; and (3) SetFit AES (Krug et al., 2025), a contrastive-learning-based framework utilizing a Longformer sentence transformer for efficient long-document modeling.

As shown in Table 5, AES models differ substantially in their modeling strategies, training paradigms, and representation learning mechanisms. Several recent studies integrate Transformer encoders with graph-based modeling to capture semantic and structural relationships within essays. For example, GDBERT-Score and ET-GNN both combine contextual Transformer embeddings with graph neural networks, although ET-GNN further incorporates ensemble learning across multiple Transformer backbones. In contrast, SetFit AES focuses on computational efficiency through contrastive sentence-transformer learning and long-context modeling using Longformer.

Compared with these approaches, the proposed PaSTO-GNN framework integrates prompt-aware learning, sentence-level spatio-temporal graph modeling, sequential contextual refinement, and ordinal score prediction within a unified architecture. Unlike existing graph-based AES approaches that primarily model semantic similarity relationships, the proposed framework jointly captures semantic and temporal discourse dependencies through spatio-temporal graph construction and BiGRU-based sequential refinement. Furthermore, the incorporation of prompt-aware representations enables adaptation to heterogeneous prompt-specific scoring distributions across essays.

The experimental results presented in Table 4 demonstrate that PaSTO-GNN achieves competitive performance relative to recent AES approaches, obtaining a calibrated QWK score of 0.8329. Although ET-GNN reports a slightly higher classification-based QWK score of 0.841, its regression-based configuration achieves a QWK score of 0.829. Furthermore, the ET-GNN framework relies on an ensemble of multiple Transformer-GCN models, resulting in substantially higher architectural complexity and computational overhead. In contrast, the proposed framework achieves strong performance using a unified prompt-aware discourse modeling strategy without requiring ensemble learning. Additionally, compared with GDBERT-Score, the proposed framework achieves substantially higher scoring agreement, which may be attributed to the integration of sequential contextual refinement and prompt-aware discourse modeling in addition to graph-based structural representation learning.

### 4.6 Ablation study

To assess the contribution of each component of the proposed architecture, we conduct an ablation study by removing or modifying individual modules while keeping the remaining model structure unchanged.

The ablation study evaluates the contribution of the main architectural components of the proposed PaSTO-GNN framework. As shown in Table 6, the complete model achieves the best calibrated performance with a QWK of 0.8329, while also maintaining strong raw performance (QWK = 0.8144, RMSE = 0.6190).

**Table 6:** Ablation study results on the AES 2.0 dataset.

Model	Component Tested	QWK	PCC	RMSE	Cal-QWK	ΔQWK
PaSTO-GNN (Full)	Full model	0.8144	0.8168	0.6190	**0.8329**	–
PaSTO-GNN w/o LoRA	Contextual adaptation	0.7829	0.7862	0.6633	0.7976	-0.0353
PaSTO-GNN w/o ST-GNN Layers	Graph message passing	0.8101	0.8186	0.6380	0.8315	-0.0014
PaSTO-GNN w/o BiGRU	Sequential modeling	0.6430	0.6490	0.8348	0.6585	-0.1744
PaSTO-GNN w/o ST-GNN & BiGRU	Graph & sequential modeling	0.6090	0.6274	0.8541	0.6430	-0.1899
PaSTO-GNN w/o Prompt Heads	Prompt-aware scoring	**0.8167**	**0.8187**	**0.6137**	0.8307	-0.0022

Cal-QWK denotes the calibrated Quadratic Weighted Kappa after post-hoc calibration. ΔQWK indicates the change relative to the full model. Bold values indicate the best performance among the compared models for each evaluation metric.

Removing the LoRA-based adaptation leads to a noticeable reduction in performance, indicating that parameter-efficient fine-tuning helps the RoBERTa encoder better capture essay-specific linguistic characteristics while avoiding full model fine-tuning. This result highlights the importance of adapting the contextual encoder to the essay scoring task.

Disabling the spatio-temporal message-passing layers results in a slight decrease in performance, indicating that explicit neighborhood aggregation over temporal and semantic sentence relations provides additional contextual cues beyond the fused sentence representations alone.

The results indicate that the BiGRU sequential refinement component contributes more substantially to predictive performance than the spatio-temporal graph propagation module alone. In particular, removing the ST-GNN layers results in only a modest reduction in calibrated QWK, whereas removing the BiGRU component leads to a substantially larger performance degradation. These findings suggest that the graph component provides complementary discourse-aware structural reasoning alongside strong sequential contextual modeling rather than serving as the sole source of performance improvement.

Replacing prompt-specific heads with a shared prediction head yields a marginal increase in raw QWK, but a slightly lower calibrated QWK. This suggests that prompt-aware heads contribute more to prompt-specific calibration and score alignment than to raw predictive accuracy alone, which is reasonable given that different prompts exhibit distinct scoring distributions and evaluation criteria.

Overall, the ablation results confirm that LoRA-based contextual adaptation, sequential modeling through BiGRU, graph-based discourse representation, and prompt-aware prediction collectively contribute to the effectiveness of the proposed PaSTO-GNN framework.

### 4.7 Discussion

The experimental results demonstrate that the proposed PaSTO-GNN framework achieves strong agreement with human raters on the AES 2.0 dataset. The model achieves a QWK score of 0.8144 before calibration and 0.8329 after *post-hoc* calibration, indicating that the combination of prompt-aware representations, discourse modeling, and ordinal prediction provides an effective approach for automated essay scoring. The consistent improvement after calibration further highlights the importance of aligning predicted score distributions with prompt-specific human grading behavior.

The per-prompt evaluation further demonstrates that the proposed framework performs consistently across heterogeneous essay prompts, achieving QWK values above 0.80 for several prompts and reaching the strongest performance on the *Facial Action Coding System* prompt. These findings suggest that the prompt-aware design enables the model to better adapt to different writing tasks, scoring rubrics, and prompt-specific score distributions.

The ablation study provides further insight into the contribution of the main architectural components. Removing LoRA adaptation results in the largest performance degradation, confirming that parameter-efficient fine-tuning of the contextual encoder plays a critical role in adapting language representations to the essay scoring task. In contrast, disabling the spatio-temporal message-passing mechanism leads to a smaller but consistent decrease in performance, indicating that while contextual sentence embeddings already capture strong semantic information, graph-based message passing provides complementary discourse-level structural reasoning that further improves essay representation quality.

Interestingly, although removing the BiGRU sequential refinement module produces a larger performance degradation than disabling the spatio-temporal message-passing mechanism alone, the joint removal of both the graph message-passing and sequential refinement components leads to an even larger reduction in performance. This observation suggests that the sequential and graph-based components provide complementary contributions to essay representation learning. Specifically, the BiGRU module contributes more substantially to contextual sequence modeling, while the graph message-passing mechanism provides additional discourse-aware structural information that further enhances representation quality when integrated with sequential refinement. These findings further highlight the complementary role of the different architectural components in the proposed framework. The LoRA-adapted RoBERTa encoder captures contextual linguistic representations while maintaining parameter efficiency, SBERT embeddings provide stable semantic similarity estimation for graph construction, and the spatio-temporal graph module incorporates discourse-aware structural relationships among sentences. In addition, prompt-aware ordinal prediction enables the model to better adapt to heterogeneous prompt-specific scoring distributions. We acknowledge that the performance improvement over strong Transformer-based baselines is moderate in absolute terms. However, benchmarks such as AES 2.0 are highly competitive, where even relatively small improvements in Quadratic Weighted Kappa (QWK) are generally considered meaningful. Furthermore, the proposed framework aims not only to improve predictive performance but also to provide a more structured and discourse-aware representation of essay composition through the integration of complementary sequential and graph-based modeling mechanisms.

We additionally compared the proposed framework with recent AES studies reported in the literature to better contextualize its performance relative to existing approaches. However, direct comparison with several state-of-the-art AES systems remains challenging due to differences in datasets, and evaluation protocols, particularly in cross-prompt training settings.

Overall, the results suggest that combining contextual sentence embeddings, prompt-aware modeling, and discourse-level structure learning leads to more robust and reliable essay scoring predictions. These findings highlight the importance of modeling both semantic and structural properties of essays when designing automated assessment systems.

Despite the encouraging performance, the proposed framework introduces additional architectural complexity compared with standard transformer-based AES baselines. Future work will investigate more computationally efficient discourse modeling strategies and lightweight graph reasoning mechanisms while maintaining strong scoring performance.

In addition, the current study focuses on prompt-stratified evaluation using the AES 2.0 dataset and does not explicitly investigate cross-prompt or cross-dataset generalization. Consequently, the ability of the proposed framework to transfer across unseen prompts or alternative essay scoring benchmarks remains an open research question. Future work will investigate prompt-transfer evaluation settings and broader generalization analysis across heterogeneous AES datasets.

## 5 Conclusion

This paper proposed PaSTO-GNN, a prompt-aware spatio-temporal graph neural network for automatic essay scoring. The framework integrates contextual sentence representations from a LoRA-adapted RoBERTa encoder with SBERT embeddings and models discourse relationships using a spatio-temporal graph architecture combined with ordinal prediction via CORAL. Experimental results on the AES 2.0 dataset demonstrate strong agreement with human raters, achieving a QWK of 0.8144 and improving to 0.8329 after calibration. The ablation study confirms the contribution of key components of the framework, particularly contextual adaptation and discourse-aware modeling, highlighting the effectiveness of combining prompt conditioning with structured essay representations.

## Data Availability

Publicly available datasets were analyzed in this study. This data can be found here: https://www.kaggle.com/datasets/lburleigh/asap-2-0.
